# Mutations of human DNA topoisomerase I at poly(ADP-ribose) binding sites: modulation of camptothecin activity by ADP-ribose polymers

**DOI:** 10.1186/s13046-014-0071-z

**Published:** 2014-09-17

**Authors:** Cinzia Tesauro, Grazia Graziani, Barbara Arnò, Laura Zuccaro, Alessia Muzi, Ilda D’Annessa, Elettra Santori, Lucio Tentori, Carlo Leonetti, Paola Fiorani, Alessandro Desideri

**Affiliations:** Department of Biology and Interuniversity Consortium, National Institute Biostructure and Biosystem (INBB), University of Rome ‘Tor Vergata’, Via della Ricerca Scientifica, Rome, 00133 Italy; Department of Systems Medicine, University of Rome Tor ‘Vergata Via’ Montpellier 1, Rome, 00133 Italy; Experimental Chemotherapy Laboratory, Regina Elena Cancer Institute, Via delle Messi d’Oro 156, Rome, 00158 Italy; Institute of Translational Pharmacology, National Research Council, CNR, Via del Fosso del Cavaliere 100, Rome, 00133 Italy

**Keywords:** Topoisomerase I, PARylation, Cleavage, Religation rate, Camptothecin, PARP inhibitors

## Abstract

**Background:**

DNA topoisomerases are key enzymes that modulate the topological state of DNA through the breaking and rejoining of DNA strands. Human topoisomerase I belongs to the family of poly(ADP-ribose)-binding proteins and is the target of camptothecin derived anticancer drugs. Poly(ADP-ribosyl)ation occurs at specific sites of the enzyme inhibiting the cleavage and enhancing the religation steps during the catalytic cycle. Thus, ADP-ribose polymers antagonize the activity of topoisomerase I poisons, whereas PARP inhibitors increase their antitumor effects.

**Methods:**

Using site-directed mutagenesis we have analyzed the interaction of human topoisomerase I and poly(ADP-ribose) through enzymatic activity and binding procedures.

**Results:**

Mutations of the human topoisomerase I hydrophobic or charged residues, located on the putative polymer binding sites, are not sufficient to abolish or reduce the binding of the poly(ADP-ribose) to the protein. These results suggest either the presence of additional binding sites or that the mutations are not enough perturbative to destroy the poly(ADP-ribose) interaction, although in one mutant they fully abolish the enzyme activity.

**Conclusions:**

It can be concluded that mutations at the hydrophobic or charged residues of the putative polymer binding sites do not interfere with the ability of poly(ADP-ribose) to antagonize the antitumor activity of topoisomerase I poisons.

## Background

Poly(ADP-ribosyl)ation (PARylation) is a post-translational modification of proteins catalyzed by the poly(ADP-ribose) polymerase (PARP) family of enzymes, of which PARP-1 is the best characterized member [1]. The ADP-ribose monomers are cleaved by PARP enzymes from NAD^+^ donor and joined together to form long and branched polymers (PARs) that are covalently linked to glutamic, aspartic and lysine amino acids of target proteins, such as histones, transcription factors and PARP-1 itself, which is the main acceptor of PARs. Through PAR-mediated auto-modification and heteromodification of proteins, PARP-1 regulates a number of cellular processes including DNA damage detection/repair, chromatin remodeling, transcription control, differentiation and cell death [2]. Covalently attached PARs have a rapid turn-over, since they are quickly hydrolyzed to free PARs or mono(ADP-ribose) by PAR glycohydrolase (PARG) [3].

PARylated PARP-1 or free PARs, that are highly flexible polymers [4], can also non-covalently interact with a variety of proteins containing a PAR binding motif (PBM), which includes basic and hydrophobic residues downstream of a positively charged cluster rich in lysine and arginine [5-8]. This motif has been found in proteins involved in DNA damage response, chromatin structure, replication and transcription and can be present in different domains of the same target protein.

Other motifs or domains capable of interacting with PARs comprise: *i*) the glycine and arginine rich domain (GAR) lacking hydrophobic amino acids, identified in proteins involved in RNA metabolism and in some chromatin associated proteins; *ii*) the RNA recognition motif (RRM) and the serine/arginine repeats (SR) domain, both present in proteins implicated in nucleic acid metabolism; *iii*) the PAR-binding zinc finger (PBZ), found only in three human proteins related to PAR metabolism; *iv*) the WWE domain, in which tryptophan (W) and glutamate (E) are the most conserved amino acids and that has been reported in proteins associated with ubiquitylation and PARylation; *v*) the macro domain, present in some PARPs, histone variants and capable of binding to PARs or ADP-ribose and O-acetyl-ADP-ribose [9]. Since PARs are negatively charged molecules, they profoundly alter the biochemical properties of the covalently-modified acceptors or proteins non-covalently interacting with PARs, modulating their structure, function and localization.

Human topoisomerase I (hTop1) has been identified as a protein that non-covalently interacts with PARs [10]. HTop1 is composed of 765 amino acids and four different domains: the NH2-terminal (residues 1–214), the core (215–635), the linker (636–712), and the COOH-terminal domain (713–765) [11]. The enzyme relaxes a supercoiled DNA substrate cleaving one strand, facilitating the rotation of one strand over the other and religating the cleaved strand after relaxation is occurred. HTop1 is of significant medical interest being the target of antitumor agents derived from camptothecin (CPT). In particular, irinotecan is approved with 5-fluorouracil and leucovorin for the first line treatment of metastatic colorectal cancer and as monotherapy for the treatment of disease recurrence or progression following fluorouracil-based therapy [12]. Topotecan, instead, is currently used for the treatment of metastatic ovarian cancer and small cell lung cancer after failure of first-line chemotherapy.

CPT reversibly binds to the covalent intermediate DNA-enzyme, stabilizing the cleavable complex and reducing the rate of religation. The stalled hTop1 complex collides with the progressing replication fork producing lethal double-stranded DNA breaks and cell death [13]. *In vitro* both the cleavage and religation reactions can be modulated by PARs [10]. In detail, DNA cleavage is inhibited, whilst the religation activity is enhanced, even when the enzyme is stalled with the CPT drug, in presence of PARs. Therefore, PARs counteract the effects of hTop1 poisons. Accordingly, PARP inhibitors remove the antagonistic effect exerted by PARs on the mechanism of action of hTop1 poisons, increasing the formation of persistent DNA breaks. Indeed, the combination of PARP inhibitors with the CPT derivatives irinotecan or topotecan resulted in synergistic antitumor effects in preclinical tumor models and is under evaluation in clinical trials for the treatment of a number of refractory malignancies [14-19] (www.clinicaltrials.gov).

**Figure 1:**
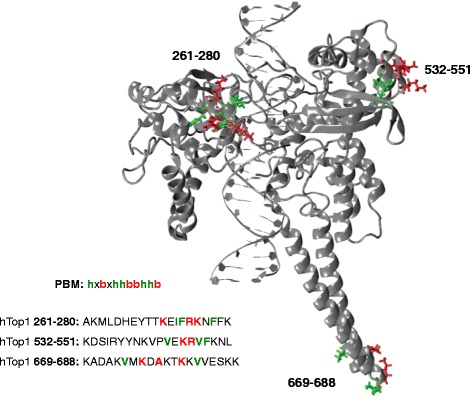
**Three-dimensional representation of the hTop1 protein-DNA binary complex.** The lateral chains of the residues forming the three putative PAR binding sites have been mapped on the hTop1 structure. The positively charged residues are shown in red and the hydrophobic ones in green.

Three putative PBM, supposed to be present in hTop1, have been identified, two of them are positioned in the core DNA-binding domain (amino acids 261–280 and 532–551, respectively), whereas the third one is in the linker domain (amino acids 669–688) that connects the core with the C-terminal domain where the catalytic tyrosine is located (Figure 1) [10]. However it has never been demonstrated that they are indispensable for the PAR binding or if they are selectively involved in the modulation of the cleavage and religation activity. In this study, in the effort to identify the functional role of PAR binding sites, we have produced two hTop1 mutants where eight basic (8bmut) and eight hydrophobic (8hmut) residues present in the three PMB have been eliminated and replaced with neutral alanines, in order to test the binding of PARs and the resulting modulation of hTop1 activity in the absence or in the presence of CPT. The basic residues were selected because they can be important in mediating an electrostatic interaction with the negatively charged PARs, whilst hydrophobic residues can have a crucial role in defining the conformation of the motif. The results show that the two mutants still bind PARs, indicating either the presence of additional PAR binding sites or that the drastic mutations are not enough to destroy the PAR interaction.

## Methods

### Yeast strains, plasmids and purification

Anti-FLAG M2 monoclonal affinity gel, FLAG peptide, anti-FLAG M2 monoclonal antibody were purchased from Sigma-Aldrich and the antibody against the C-terminus of hTop1 from Abcam.

*Saccharomyces cerevisiae* Top1 null strain EKY3 (ura3-52, his3Δ200, leu2Δ1, trp1Δ63, top1::TRP1, MATα) was used to express the hTop1 gene. YCpGAL1-e-hTop1 single copy plasmid was previously described [20].

The 8bmut and the 8hmut mutants were generated using a site-directed-mutagenesis kit (Agilent Technologies) of the YCpGAL1-hTop1 in which the hTop1 is expressed under the galactose inducible promoter in a single-copy plasmid. The epitope-tagged construct YCpGAL1-e-hTop1 contains the N-terminal sequence FLAG: DYKDDDDY (indicated with ‘e’), recognized by the M2 monoclonal antibody. The epitope-tag was subcloned into YCpGAL1-hTop18bmut or YCpGAL1-hTop18hmut to produce the YCpGAL1-e-hTop18bmut and YCpGAL1-e-hTop18hmut. The plasmids were transformed into XL10-Gold *E. coli* cells (Agilent Technologies) and, then, extracted using Quiagen miniprep kit. Positive clones were identified by sequencing the hTop1 gene of the extracted plasmids. After the transformation in EKY3 yeast strain, the purification of hTop1 proteins was carried out essentially as previously described [21].

### Relaxation assay

The activity of equal amounts of wild-type or 8bmut proteins was assayed in 30 μl of reaction volume containing 0.5 μg of negatively supercoiled pBlue-Script KSII(+) DNA, which is present in both dimeric and monomeric forms, and reaction buffer (20 mM Tris–HCl pH 7.5, 0.1 mM Na_2_EDTA, 10 mM MgCl_2_, 5 μg/ml acetylated bovine serum albumin and 150 mM KCl). The effect of PARs on hTop1 enzymatic activity was measured by adding increasing pmol of PARs to the reactions that were stopped with 0.5% SDS after 10 minutes at 37°C. In selected samples no proteins have been added as negative control or no PARs have been added to show the full capability of relaxation of the proteins. The samples were resolved in a 1% (w/v) agarose gel in 48 mM Tris, 45.5 mM boric acid, 1 mM EDTA at 10 V/cm. The gels were stained with ethidium bromide (0.5 μg/ml), destained with water and photographed using an UV transilluminator.

### Synthesis *in vitro* and analysis by gel electrophoresis of [^32^P]-PARs

Polymers of [^32^P]ADP-ribose, with high specific activity (0.5 μCi/nmol), were synthesized in a 100 μl reaction mixture containing 100 mM Tris–HCl pH 8, 10 mM MgCl_2_, 2 mM dithiothreitol, [^32^P]-NAD^+^ (1000 Ci/mmol; 5 μCi/μl; GE Healthcare), 1 μg of purified PARP-1 (Trevigen), 2.5 μg of DNase I-activated DNA (Alexis Biochemicals) and 200 μM NAD^+^ (Sigma-Aldrich). After 45 min incubation at 30°C the reaction was stopped by the addition of ice-cold trichloroacetic acid and PARs were detached from the proteins as described [22]. The concentration of radiolabeled PARs was expressed as nmol monomeric [^32^P]ADP-ribose/μl and calculated as previously described (dpm μl/NAD-specific radioactivity) [22]. For the synthesis of cold PARs the radiolabeled NAD^+^ was omitted from the reaction mixture.

An aliquot of radiolabeled PARs (corresponding to 25,000 dpm) was loaded onto a 20% polyacrylamide gel and electrophoresed until bromophenol blue (co-migrating with the 8-mer of ADP-ribose) reached half the length of the gel [22,23]. Gels were dried under vacuum and exposed to X-ray films (Kodak X-Omat).

### Dot blot analysis of wild-type and hTop1 mutants

Purified wild-type and hTop1 recombinant mutants were quantified by dot blot analysis using an antibody against the C-terminal region of hTop1 or the anti-FLAG antibody conjugated with alkaline phosphatase (clone M2, Sigma-Aldrich). In the case of immunoblot with the anti-hTop1 antibody, the membrane was, then, incubated with goat anti-mouse IgG horseradish peroxidase (HRP)-conjugated secondary antibody and immunoreactive bands were detected by enhanced chemoluminescence (ECL) technique using the ECL Plus Western Blotting Substrate (Pierce). For the staining of immunoreactive bands of the membranes incubated with the anti-FLAG antibody, the NBT/BCIP substrates and solutions were used (Sigma-Aldrich). Spots were quantified by Image J densitometry software (National Institutes of Health, Bethesda, MD, http://rsbweb.nih.gov/ij/).

### PAR binding assay

Graded amounts of purified wild-type and hTop1 mutants were spotted on nitrocellulose membrane (0.45 μm; GE healthcare). Histone H1 was used as positive control in the PAR binding assay, whereas DNase I and proteinase K were used as negative controls. Non-covalent binding of PARs to hTop1 immobilized on nitrocellulose membrane was assayed incubating the filter with radiolabeled PARs (0.5 nmol) diluted in 10 ml of TBS-T (10 mM Tris–HCl pH 8.0, 150 mM NaCl, 0.1% Tween 20) for 1 h at room temperature, as described [10]. The membrane was washed with TBS-T buffer, until no radioactivity was detected in the last wash, and exposed to X-ray film.

### Cleavage and religation assay

Oligonucleotide CL14 (5′-GAAAAAAGACTTAG-3′) that contains a hTop1 high affinity cleavage site was 5′-end labeled with [γ^32^P]-ATP. The CP25 complementary strand (5′-TAAAAATTTTTCTAAGTCTTTTTTC-3′) was 5′-end phosphorylated with unlabeled ATP. The two strands were annealed with a 2-fold molar excess of CP25 over CL14. The suicide cleavage reactions were carried out, as a function of the indicated PAR concentrations, by incubating 20 nM of the duplex DNA with an excess of hTop1 or the 8bmut in 10 mM Tris pH 7.5, 5 mM MgCl_2_, 5 mM CaCl_2_ and 150 mM KCl at 25°C in a final volume of 50 μl [24]. At various time points 5 μl aliquots were removed and the reaction stopped with 0.5% (w/v) SDS. After ethanol precipitation samples were resuspended in 5 μl of 1 mg/ml trypsin and incubated at 37°C for 60 minutes. However, a short trypsin resistant peptide is always left explaining why the cleavable complex (Cl) migrates slower than the uncleaved [25]. Samples have been analyzed by denaturing 7 M urea/20% polyacrylamide gel electrophoresis in TBE (48 mM Tris, 45.5 mM Boric Acid, 1 mM EDTA). The percentage of cleaved substrate was determined by PhosphorImager and ImageQuant software, normalized on the total amount of radioactivity in each lane and finally plotted as a function of PAR concentrations.

For the religation assay, 20 nM of CL14/CP25 (radiolabeled as previously described in the cleavage kinetic experiment) was incubated with an excess of hTop1 or 8bmut for 60 minutes at 25°C followed by 30 minutes at 37°C in 20 mM Tris–HCl pH 7.5, 0.1 mM Na_2_EDTA, 10 mM MgCl_2_, 50 μg/ml acetylated BSA, and 150 mM KCl. After the formation of the cleavable complex a 5 μl aliquot was removed and used as time 0 point, then DMSO, 100 μM CPT, PARs or CPT plus PARs were added and religation reaction was started by adding a 200-fold molar excess of R11 oligonucleotide (5′-AGAAAAATTTT-3′) over the CL14/CP25 [22]. Five μl aliquots were removed at various time points, and the reaction stopped with 0.5% SDS. After ethanol precipitation, samples were resuspended in 5 μl of 1 mg/ml trypsin and incubated at 37°C for 60 minutes. Samples were analyzed by denaturing 7 M urea/20% polyacrylamide gel electrophoresis in 48 mM Tris, 45.5 mM boric acid, 1 mM EDTA. The percentage of remaining cleavage complex was quantified by ImageQuant software, normalized to the total radioactivity for each lane and to the value at t = 0 and finally plotted as a function of time.

## Results and discussion

**Figure 2:**
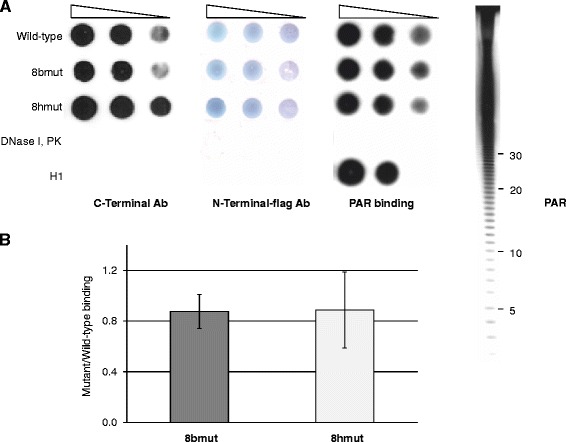
**Non-covalent PAR binding of wild-type hTop1, 8bmut and 8hmut. A**. The wild-type hTop1, 8bmut and 8hmut were spotted on nitrocellulose membrane and quantified by immunoblot analysis using anti-hTop1 C-terminal antibody or N-terminal-FLAG antibodies. The same graded amounts of proteins were tested for non-covalent PAR binding using radiolabeled PARs. Negative (1 μg DNase I and 1 μg proteinase K, PK) and positive (50 and 25 ng histone H1) (H1) controls have been also spotted on the membrane. The typical ladder of [^32^P]-PARs is shown on the right and the length of PAR molecules in terms of ADP-ribose residues is indicated. **B**. Bars represent the ratio of the PAR binding of the mutants and the wild-type protein after normalization with anti-hTop1 (C-terminus) or N-terminal-FLAG antibodies. The values are the means ± SD of three independent experiments.

The three putative PBM, which are present in the core and linker domains of hTop1 (Figure 1), contain eight basic amino acids (in red), likely important in mediating an electrostatic interaction with the negatively charged PARs, and eight hydrophobic amino acids (in green) that might have a crucial role in defining the conformation of the motif. Two mutants, one replacing the eight basic (8bmut) and the other one the eight hydrophobic (8hmut) amino acids with neutral alanines have been produced by site directed mutagenesis. Both mutants and the wild-type protein have been introduced in the single copy yeast plasmid (YCp) expressing the hTop1 under the GAL1 promoter. The PAR binding has been tested using a nitrocellulose blot assay. Serial dilutions of the two mutants and the wild-type proteins have been spotted on the nitrocellulose filter (Figure 2A), and the non-covalent binding of radiolabeled PARs, added to the filter, has been quantified after washing. The detection of the radioactive signal in the three samples indicates that the mutants retain the ability to bind PARs, as confirmed by the negative (DNase I and proteinase K) and positive (histone H1) controls. The quantitative analysis of the results of PAR binding assays from three independent experiments, after normalization with the amount of hTop1 spotted on the filter, shows that the PAR binding ability of the two mutants is similar to that of the wild-type protein (Figure 2B).

**Figure 3:**
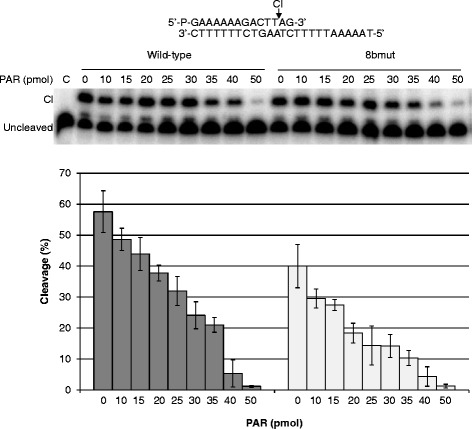
**Influence of PARs on cleavage reaction.** Suicide cleavage reaction as a function of PAR concentration, carried out with the substrate described on the top of the figure. Different concentrations of PARs were incubated with the wild-type or 8bmut proteins at 37°C for 30 min. Cl represents the DNA fragment cleaved by the enzyme at the preferred site. Lane 1, no protein added. The percentage of cleaved suicide substrate has been plotted against different PAR concentrations for the wild-type (dark grey bar) and the 8bmut (light gray bar). Data shown are means ± SD from 3 independent experiments.

Analysis of the specific activity of the purified hTop1, of the 8bmut and of the 8hmut determined using a plasmid DNA relaxation assay indicates that the 8bmut has an activity comparable to that of the wild-type protein, whilst the 8hmut protein is completely inactive (data not shown). The loss of activity of the 8hmut does not permit to measure the effect of PARs on the cleavage and religation activity and indicates that elimination of eight hydrophobic residues induces a conformational change that is enough to inactivate the protein but not to eliminate the PAR binding. The cleavage of the wild-type and 8bmut enzymes was analyzed in the presence or in the absence of graded concentrations of PARs using a suicide cleavage substrate (Figure 3). In detail, a 5^′^-end radiolabeled oligonucleotide CL14 (5^′^-GAAAAAAGACTT′AG-3^′^) has been annealed to the CP25 (5^′^-TAAAAATTTTTCTAAGTCTTTTTTC-3^′^) complementary strand, to produce a duplex with an 11-base 5^′^-single-strand extension. The enzyme cuts preferentially at the site indicated by the arrow and the religation step is precluded because the AG-3^′^ oligonucleotide is too short to be religated. Therefore, the enzyme remains covalently attached to the 3^′^-end and becomes trapped in the covalent complex, permitting to measure the protein cleavage efficiency by quantification of the band corresponding to the cut DNA, because of the different migration when compared to the uncleaved one (Figure 3). The results indicate that, although the mutant protein has a cutting efficiency 20% lower than that of the wild-type protein, the cleavage reaction is reduced by PARs in a dose-dependent manner. Noteworthy, 50 pmol PARs almost abrogate the cleavage of both the 8bmut and the wild-type proteins.

**Figure 4:**
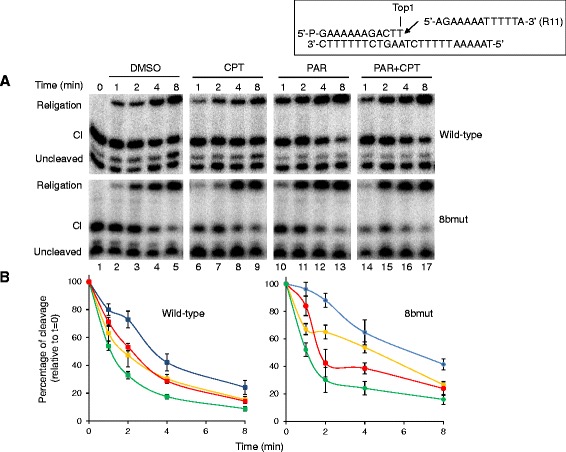
**Influence of PARs on the kinetics of religation. A**. Time course (1–8 min) of the religation experiment between the complementary R11 DNA strand and the enzyme covalent complexes described at the top of the figure, in the absence (lanes 2–5) or presence of 100 μM CPT (lanes 6–9) or 50 pmol PARs (lanes 10–13) and PARs plus CPT (lanes 14–17). Lane 1: no R11 added; lane 18: no protein added. Cl represents the DNA fragment cleaved at the preferred site. **B**. Plots of the percentage of disappearance of the cleavage complex relative to time 0, for the wild-type (square symbol) and 8bmut (circle symbols), in the absence (yellow lines) or presence of CPT (blue lines), PARs (green lines) and PARs plus CPT (red lines). Data shown are means ± SD from 3 independent experiments.

The DNA religation step has been studied testing the ability of the wild-type protein and the 8bmut to religate the R11 (5^′^-AGAAAAATTTT-3^′^) oligonucleotide added to the cleaved suicide substrate previously incubated with an excess of enzyme. The religation has been analyzed in the presence of DMSO, PARs, CPT or PARs plus CPT. Aliquots have been removed at different times, the reaction stopped by addition of SDS and the products analyzed by PAGE electrophoresis (Figure 4A). The percentage of the remaining covalent complex (Cl), normalized to the value at t = 0, is plotted as a function of time in Figure 4B. The results show that the 8bmut has a slower religation rate compared to the wild-type protein (lanes 2–5), as expected due to the introduction of eight different amino acid substitutions. Anyhow, the addition of PARs to the reaction mixture enhances the religation rate similarly to the wild-type protein (lanes 10–13). CPT inhibits in both enzymes the religation step (lanes 6–9) that is restored in presence of PARs (lanes 14–17), as also shown by the quantitative plot of Figure 4B. These findings are consistent with the antagonistic effects exerted by PARs on the activity of hTop1 poisons [10].

Overall these data indicate that the mutation of eight hydrophobic or of eight charged residues present in the three hTop1 putative PBM does not abolish the PAR-protein interaction, and that in the case of the 8bmut, that retains the enzymatic activity, PARs inhibit the cleavage and enhance the religation identically as in the wild-type protein. This implies either the presence of additional PAR binding sites or that the drastic mutations are not able to abolish the PAR-protein interaction, even though, in the case of the 8hmut, they fully abolish the enzymatic activity. A study carried out on 22 amino acids long peptides, containing the supposed hTop1 PAR binding motif with the same sequences of the ones reported in Figure 1, revealed the absence of any interaction with PAR [26], suggesting that these peptides do not represent the PAR binding motifs. However, a careful bioinformatics analysis of the protein sequence and structure does not provide any evidence for the presence of additional known PAR binding sites. Our results show that the native protein efficiently binds PAR, indicating the importance to study the interaction in the full protein. In addition, the protein still efficiently binds PAR even after the very strong perturbation due to the elimination of the hydrophobic or charged residues able, in the case of the 8hmut, to fully abolish the enzyme function, leaving open the question whether the protein contains additional unknown PAR binding sites.

## Conclusions

PARP inhibitors, such as veliparib, olaparib and BMN-673, are currently in phase 1 and 2 clinical trials in combination with irinotecan and topotecan to assess their safety and efficacy [www.clinicaltrials.gov]. Our data suggest that the presence of mutations at eight basic amino acids of the three putative PBM does not interfere with the ability of PARs to exert antagonistic effects on hTop1 poisons and, indirectly, with the chemosensitizing effects exerted by PARP inhibitors.

## Abbreviations

PARP: Poly-ADP-ribose polymerase
PARs: Poly(ADP-ribose)
PBM: PAR binding motif
hTop1: Human topoisomerase I
CPT: Camptothecins
